# Iodine accumulation of the liver in patients treated with amiodarone can be unmasked using material decomposition from multiphase spectral-detector CT

**DOI:** 10.1038/s41598-020-64002-7

**Published:** 2020-04-24

**Authors:** Kai Roman Laukamp, Simon Lennartz, Ahmad Hashmi, Markus Obmann, Vivian Ho, Nils Große Hokamp, Frank Philipp Graner, Robert Gilkeson, Thorsten Persigehl, Amit Gupta, Nikhil Ramaiya

**Affiliations:** 1 University Hospitals Cleveland Medical Center, Department of Radiology, Cleveland, OH USA; 2 Case Western Reserve University, Department of Radiology, Cleveland, OH USA; 3 Institute for Diagnostic and Interventional Radiology, Faculty of Medicine and University Hospital Cologne, University of Cologne, Cologne, Germany; 4 Department of Radiology, Massachusetts General Hospital, 55 Fruit St, White 270, Boston, MA 02114 USA; 5 University Hospital Basel, Department of Radiology and Nuclear Medicine, Basel, Switzerland

**Keywords:** Liver, Hepatology

## Abstract

Amiodarone accumulates in the liver, where it increases x-ray attenuation due to its iodine content. We evaluated liver attenuation in patients treated and not treated with amiodarone using true-non-contrast (TNC) and virtual-non-contrast (VNC) images acquired with spectral-detector-CT (SDCT). 142 patients, of which 21 have been treated with amiodarone, receiving SDCT-examinations (unenhanced-chest CT [TNC], CT-angiography of chest and abdomen [CTA-Chest, CTA-Abdomen]) were included. TNC, CTA-Chest, CTA-Abdomen, and corresponding VNC-images (VNC-Chest, VNC-Abdomen) were reconstructed. Liver-attenuation-index (LAI) was calculated as difference between liver- and spleen-attenuation. Liver-attenuation and LAI derived from TNC-images of patients receiving amiodarone were higher. Contrary to TNC, liver-attenuation and LAI were not higher in amiodarone patients in VNC-Chest and in VNC-Abdomen. To verify these initial results, a phantom scan was performed and an additional patient cohort included, both confirming that VNC is viable of accurately subtracting iodine of hepatic amiodarone-deposits. This might help to monitor liver-attenuation more accurately and thereby detect liver steatosis as a sign of liver damage earlier as well as to verify amiodarone accumulation in the liver.

## Introduction

Amiodarone is an antiarrhythmic drug that has shown its effectiveness for several decades to treat various forms of tachyarrhythmia^1,2^. However, oral absorption and bioavailability are poor and greatly variable. Onset of therapeutic effects using oral application range between 2–21 days and after long-term treatment plasma half-life is around 100 days. Because of its lipophilic properties, iodine-containing amiodarone accumulates, among others, in hepatocytes. Here, it inhibits the enzyme phospholipase which subsequentially stops the removal of lysosomal phospholipids. Consequently, amiodarone forms a nondigestible complex with the phospholipids, resulting in phospholipidosis and extended amiodarone accumulation. Amiodarone concentrations in the liver can be up to 500-times higher than in the blood serum and release from hepatocytes is slow after treatment discontinuation^1–6^. Earlier studies found that this condition can lead to steatohepatitis and liver cirrhosis^1,7,8^.

Iodinated contrast media is broadly applied in CT imaging. Due to its high physical density and k-edge effects of iodine, it increases attenuation and image contrast that improves depiction of anatomical structures (e.g. organ parenchyma and vessels)^9,10^. For intravascular injections in CT imaging almost exclusively non-ionic low- or iso-osmotic iodinated contrast media is applied. Molecular structures between agents are similar, a commonly applied agent is for example Ioversol (C_18_H_24_I_3_N_3_O_9_, molecular mass of 807.1 g/mol). It contains three iodine atoms that constitute almost 50% of its molecular mass^9–12^. Amiodarone shows several similarities to iodinated contrast media regarding the molecular structure. It is a di-iodinated benzofuran derivate, as a drug it is usually orally delivered as amiodarone hydrochloride (C_25_H_30_ClI_2_NO_3_, molecular mass of 681.8 g/mol). The two iodine atoms are also attached to a benzene ring and constitute almost 40% of its molecular mass^13–16^. As amiodarone tends to accumulate in the liver as alluded to above and shows a comparable atomic composition as iodinated contrast, attenuation or image contrast of the liver should increase similarly as with iodinated contrast media^17^. Accordingly, several studies showed that patients treated with amiodarone showed increased attenuation values of the liver in CT imaging^13,18,19^, that normalize slowly after treatment discontinuation^1,7,20^.

Dual-energy CT uses different technical approaches to sperate attenuation of low and high energetic photons enabling the reconstruction of virtual monoenergetic images and material decomposition. Most technical approaches are emission-based solutions such as dual-spin, dual-helix, dual-source, split or twin beam, and rapid kVp switching. There is only one detector-based system available, referred to as spectral-detector CT (SDCT). This scanner uses one X-ray source and a detector with two layers; photons with higher energies are detected in the lower layer, while lower energetic photons are detected in the upper layer^21,22^. Material decomposition can be used for the identification of iodine and thereby the creation of virtual non-contrast images (VNC) that offer a way to eliminate the iodine-associated attenuation from images. Usually this technique is used to remove iodine resulting from contrast media administration but it should also be able to remove iodine bound to amiodarone^21,23–25^. Although recently suggested^13^, no study investigated the application of dual-energy CT for subtraction of the iodine in amiodarone patients.

In our institution a cohort of patients received a dedicated CT-protocol for transcatheter-aortic-valve-replacement (TAVR) planning^26,27^, that included an unenhanced examination of the chest and CT angiographies (CTA) of the chest as well as the abdomen. In this patient cohort, several patients received amiodarone treatment. We therefore wanted to investigate: (i) the influence of amiodarone on liver attenuation in unenhanced CT acquisitions and (ii) the potential of spectral-detector CT for quantitative liver attenuation by evaluating accuracy of VNC to subtract iodine from iodinated contrast media and amiodarone. To confirm our primary analysis, an additional phantom scan and an additional patient cohort were included to confirm our initial findings.

## Results

The 142 included patients comprised 58 women and 63 men with a mean age of 81.2 ± 8.6 years, ranging from 56–97 years. In total, five patients showed signs of severe liver steatosis indicated by a reduced liver attenuation (<40 HU) and/or reduced liver attenuation index (LAI, <−10 HU). The 21 patients treated with amiodarone received the fowling daily dosages: 100 mg/day, n = 1; 200 mg/day, n = 15, 400 mg/day, n = 5. Of these 21 patients 9 were woman, while 12 were men with a mean age of 77.0 ± 10.7 years, ranging from 56–99 years (for more detailed information see Table 1). There was no statistical difference in distribution between patients receiving amiodarone treatment compared to the patients not receiving amiodarone treatment regarding body mass index, history of smoking, and presence of diabetes, arterial hypertension, abnormal liver laboratory tests, as well as chronic liver disease.

**Table 1:** Amiodarone Patients.

Patient	Gender	Age	Amiodarone dose per day [mg]	Treatment duration [months]	Atrial fibrillation
1	male	87	200	11	yes
2	female	56	200	n/a	yes
3	male	61	400	5	no
4	male	99	200	59	no
5	female	77	200	2	yes
6	male	67	200	3	yes
7	male	68	400	2	yes
8	female	67	200	11	no
9	female	89	200	54	yes
10	male	70	200	n/a	yes
11	female	69	400	n/a	yes
12	female	85	200	3	yes
13	male	68	100	21	yes
14	male	78	200	n/a	yes
15	male	75	200	20	no
16	male	86	400	n/a	yes
17	male	89	200	4	yes
18	female	77	200	3	yes
19	female	87	400	n/a	yes
20	male	78	200	4	yes
21	female	82	200	n/a	yes

LFT - Liver function test, n/a - not available.

### Objective assessment

**Figure 1:**
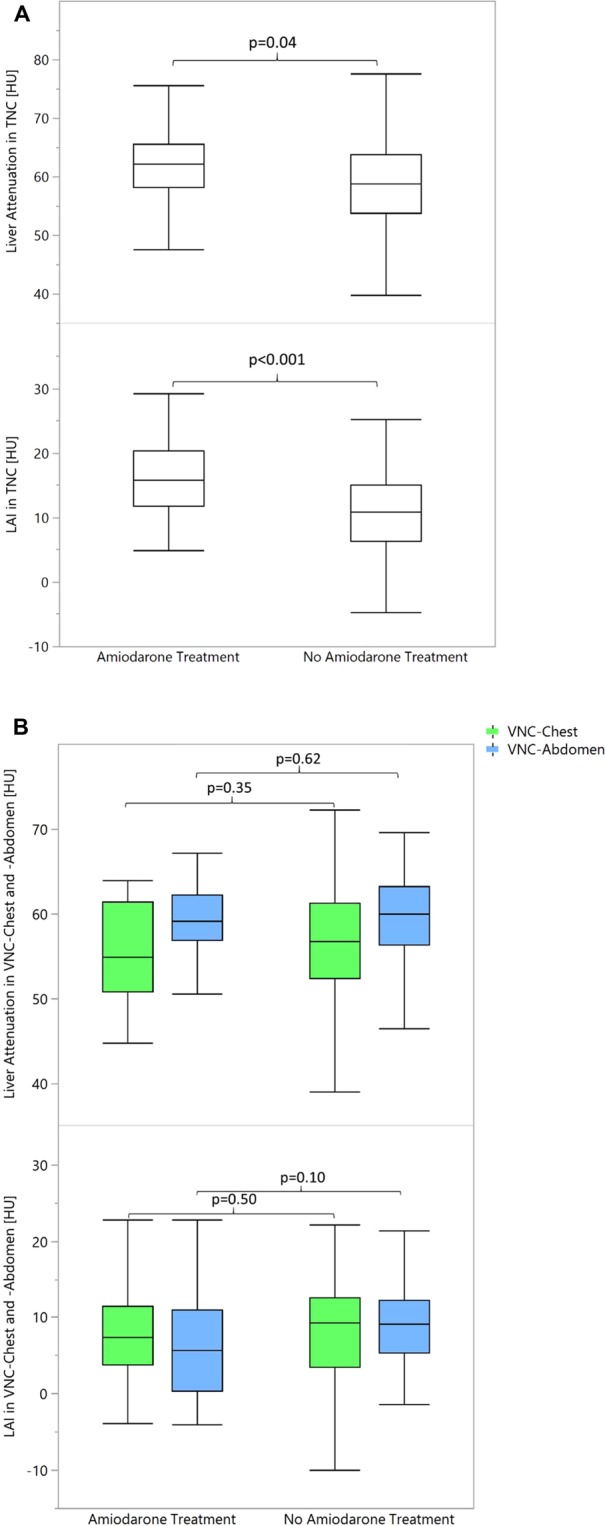
Box-plot diagram displaying attenuation in liver and liver attenuation index (LAI) in **(A)** true non-contrast (TNC), **(B)** virtual non-contrast (VNC) from CT angiography of the chest (VNC-Chest) and abdomen (VNC-Abdomen). **(A)** Patients receiving amiodarone treatment showed significantly higher liver attenuation and LAI indicating that amiodarone accumulation in the liver artificially increased attenuation values of the liver. **(B)** Contrary to TNC, liver attenuation and LAI in VNC-Chest and -Abdomen images of patients treated with amiodarone were not higher compared to patients that have not been treated with amiodarone indicating that VNC not only subtracts iodine from contrast media but might also be able to subtract the iodine in amiodarone accumulated in the liver.

**Figure 2:**
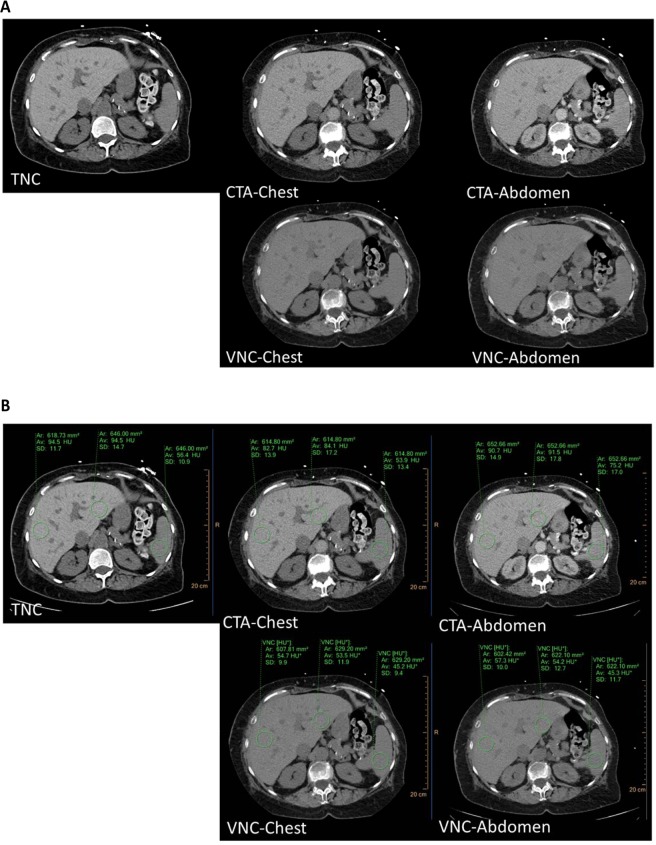
**(A)** Axial CT images in soft tissue window settings of the upper abdomen in a 72-year-old female receiving TAVR planning examination. The patient was treated with amiodarone 200 mg/day for nine months prior to imaging. The examination was conducted in February 2018. Depicted are true non-contrast (TNC), CT angiography (CTA) of the chest and abdomen (CTA-Chest and -Abdomen), as well as virtual non-contrast (VNC) from CTA-Chest (VNC-Chest) and CTA-Abdomen (VNC-Abdomen). Attenuation of the liver is increased in TNC (~95 HU) which is likely to be caused by amiodarone treatment and accumulation in the liver. Liver attenuation is noticeably lower in VNC-Chest and -Abdomen (~54–57 HU) compared to TNC (~95 HU) indicating that VNC not only subtracts iodine from contrast media but might also be able to subtract iodine from amiodarone deposits. However, attenuation in the spleen (where amiodarone usually does not accumulate) is comparable between VNC Chest- and -Abdomen (~45 HU) and TNC (~56 HU). **(B)** Images of Fig. 3A with additional ROI-placement.

The Shapiro-Wilk test did not show normal distribution for values in all assessed groups (p < 0.05). The 21 patients receiving amiodarone treatment showed significantly higher liver attenuation of 63.1 ± 9.8 HU on true non-contrast (TNC) images compared to 121 patients not receiving amiodarone treatment (58.1 ± 8.6 HU, p = 0.04, Table 2, Fig. 1A). See Fig. 2 as an example for elevated liver attenuation in TNC. Attenuation values in patients with amiodarone treatment ranged from 47.6 to 94.3 HU. Applying the LAI (intended to offer more robustness regarding patient specific differences in attenuation), the same difference could be observed, showing increased values of LAI in patients treated with amiodarone (16.8 ± 7.9 HU versus 9.7 ± 8.1 HU, p < 0.001, Table 3, Fig. 1A). LAI values for amiodarone treatment patients ranged from 5.0 to 38.5 HU. If all patients with definite signs of liver steatosis (n = 5) were removed liver attenuation and LAI in TNC images were still significantly higher in amiodarone patients (p < 0.05).

**Table 2:** Liver attenuation.

		Liver attenuation
All patients	TNC	58.8 ± 9.0
	VNC-Chest	56.1 ± 8.0
	VNC-Abdomen	59.1 ± 6.3
	Difference of TNC and VNC-Chest	2.7 ± 5.8
	Difference of TNC and VNC-Abdomen	−0.3 ± 6.7
Patients without Amiodarone treatment	TNC	58.1 ± 8.6
	VNC-Chest	56.3 ± 8.3
	VNC-Abdomen	59.2 ± 6.4
	Difference of TNC and VNC-Chest	1.8 ± 4.6
	Difference of TNC and VNC-Abdomen	−1.1 ± 5.6
Patients with Amiodarone treatment	TNC	63.1 ± 9.8
	VNC-Chest	55.3 ± 5.8
	VNC-Abdomen	58.7 ± 5.8
	Difference of TNC and VNC-Chest	7.7 ± 8.9
	Difference of TNC and VNC-Abdomen	4.3 ± 10.2
*p-values*		
Patients with Amiodarone treatment vs. no Amiodarone	TNC	**p = 0.04**
	VNC-Chest	p = 0.35
	VNC-Abdomen	p = 0.62
	Difference of TNC and VNC-Chest	**p = 0.002**
	Difference of TNC and VNC-Abdomen	**p = 0.004**

Attenuation in HU, LAI - Liver attenuation index, TNC - true non-contrast, VNC - virtual non-contrast, significant differences in bold.

**Table 3:** Liver attenuation index.

		Liver attenuation index	Difference of liver attenuation index between TNC and VNC
All patients	TNC	10.7 ± 8.4	n/a
	VNC-Chest	7.5 ± 8.2	3.2 ± 7.5
	VNC-Abdomen	8.2 ± 7.1	2.5 ± 6.7
Patients without Amiodarone treatment	TNC	9.7 ± 8.1	n/a
	VNC-Chest	7.5 ± 8.5	2.2 ± 6.9
	VNC-Abdomen	8.5 ± 6.5	1.1 ± 5.4
Patients with Amiodarone treatment	TNC	16.8 ± 7.9	n/a
	VNC-Chest	7.5 ± 6.9	9.2 ± 8.0
	VNC-Abdomen	6.5 ± 6.7	10.3 ± 8.6
*p-values*			
Patients with Amiodarone treatment vs. no Amiodarone	TNC	**p < 0.001**	n/a
	VNC-Chest	p = 0.50	**p < 0.001**
	VNC-Abdomen	p = 0.10	**p < 0.001**

Attenuation in HU, LAI - Liver attenuation index, TNC - true non-contrast, VNC - virtual non-contrast, significant differences in bold.

Contrary to TNC, liver attenuation in VNC-Chest and -Abdomen images of patients treated with amiodarone were not higher (VNC-Chest: 55.3 ± 5.8HU, VNC-Abdomen 58.7 ± 5.8 HU) compared to patients that have not been treated with amiodarone (VNC-Chest: 56.3 ± 8.3 HU [p = 0.35], VNC-Abdomen 59.2 ± 6.4 HU [p = 0.62], Table 2, Fig. 1B). The same trend could be seen in the LAI, as LAI values in VNC-Chest and Abdomen did not show significant differences between treated and untreated patients (amiodarone, VNC-Chest: 7.5 ± 6.9 HU, VNC-Abdomen 6.5 ± 6.7 HU; no amiodarone, VNC-Chest: 7.5 ± 8.5 HU [p = 0.50], VNC-Abdomen 8.5 ± 6.5 HU [p = 0.10], Table 3, Fig. 1B). When patients with signs of liver steatosis (n = 5) were removed, liver attenuation and LAI in VNC-Chest and -Abdomen still did not show significant differences between patients with and without amiodarone treatment (p > 0.05).

For evaluation of VNC accuracy in CTA-Chest- and -Abdomen, we excluded amiodarone patients to avoid influence of elevated liver attenuation from amiodarone accumulation in the liver. In liver and spleen, the 121 patients without amiodarone treatment showed VNC-Chest and -Abdomen attenuation values very similar to TNC (average liver attenuation: TNC, 58.1 ± 8.6HU, VNC-Chest, 56.3 ± 8.3HU [p = 0.034], VNC-Abdomen, 59.2 ± 6.4HU, [p = 0.34]; average spleen attenuation: TNC, 48.4 ± 6.1HU, VNC-Chest 48.7 ± 5.2HU [p = 0.71], VNC-Abdomen, 50.6 ± 4.2HU [p = 0.001]).

In the additional 145 TAVR patients that also received VNC reconstructions of the TNC scans, the eleven patients receiving amiodarone treatment showed significantly higher liver attenuation of 61.6 ± 13.3 HU and LAI of 18.5 ± 8.7 on TNC compared to the 134 patients not receiving amiodarone treatment (liver attenuation, 54.4 ± 8.2 HU, p = 0.025; LAI, 10.1 ± 9.1 HU, p = 0.008, Supplementary Fig. 1). Contrary, in patients treated with amiodarone in VNC applied to the TNC images liver attenuation (52.9 ± 9.3 HU) and LAI (7.7 ± 8.3 HU) were comparable without significant differences and not higher compared to patients that have not been treated with amiodarone (liver attenuation, 53.9 ± 8.3 HU, p > 0.05; LAI, 9.7 ± 8.5 HU, p > 0.05, Supplementary Fig. 1).

In the phantom scan, attenuation of the minced liver without any additions was 28.9 ± 0.9 HU in TNC and 28.6 ± 1.0 HU in VNC. The minced liver mixed with amiodarone hydrochloride displayed attenuation values of 40.7 ± 1.1 HU in TNC images; in VNC attenuation was then reduced to values comparable to minced liver without any additions (31.9 ± 0.9 HU).

## Discussion

This study quantitatively evaluated liver and spleen attenuation in patients with and without amiodarone treatment using TNC and VNC from spectral-detector CT. Patients that were being treated with amiodarone showed higher values of liver attenuation and LAI in TNC images. This is in line with previous studies^13,18,19^ and case reports^7,28,29^ suggesting a connection between amiodarone treatment and elevated liver attenuation. Besides the aforementioned case reports, there is only one recent study examining the influence of amiodarone treatment on liver attenuation in CT. The study from Matsuda *et al*. showed an increase of mean liver attenuation of 9.7 HU in 25 patients after amiodarone treatment had been initiated. This is in line with our investigation that revealed also an increase of liver attenuation by 5.0 HU and an increase of LAI by 7.1 HU. Our results and earlier studies suggest that the parenchymal attenuation increase might be caused by the iodine bound to amiodarone molecule accumulated in the liver, similar to the way iodinated contrast media increases attenuation^7,13–16,30–32^.

VNC-Chest and -Abdomen derived from spectral-detector CT in a cohort of TAVR planning patients not receiving amiodarone treatment showed accurate removal of iodine in both contrast phases (CTA-Chest and -Abdomen) with attenuation values very similar to TNC. In general, for quantitative liver assessment VNC might therefore serve as a replacement to unenhanced CT examinations when potential limitations of this technique are considered^33,34^. For example, Durieux *et al*. 2018 evaluated VNC images for abdominal depiction from a third generation dual-source dual-energy CT and pointed out that despite high accuracies of attenuation values for the liver and spleen with mean attenuation differences between 0–2 HU, there have been substantial differences in fluid, fat and renal tissue with mean differences as high as 32 HU. Other recent dual-energy studies also showed accurate iodine subtraction by VNC for depiction of the liver^23,24,35,36^. Further, overestimation of fat by VNC has also been reported for spectral-detector CT by Ananthakrishnan *et al*. and Sauter *et al*., which could negatively impact attenuation based diagnosis of pathologies with increased fat content, such as liver steatosis, as recently shown by Haji-Momeninan *et al*. in a second-generation dual-source CT scanner^23,24,35^. For depiction of the liver, Haji-Momenian *et al*. reported mean attenuation differences in liver between TNC and VNC of 5.4 ± 8.4 HU in 48 patients^24^. Our study showed differences between TNC and VNC of 1.8 ± 4.6 HU for CTA-Chest and −1.1 ± 5.9 HU in CTA-Abdomen in liver. These smaller differences in accuracy between the dual-energy CT solutions available might to some extend also be explained by their technical approaches. The spectral-detector CT, for example, separates high and low energy photons on the detector level so that acquired data are exactly matched temporally and spatially. Thereby material decomposition is enabled in the raw data domain^21,23,37,38^.

Dual-energy CT allows for identification of iodine containing voxels. In a second step, the iodine component of a predefined voxel can be measured and accurately subtracted to create VNC images. This approach should be equally valid for iodinated contrast media and iodine bound to amiodarone^21^ which have similar molecular structures. The iodine molecules are connected to a benzene ring and iodine atoms have a comparable share of the total molecular mass^9–16^. In our study, contrary to TNC, liver attenuation and LAI in VNC of CTA-Chest and -Abdomen examinations did not differ significantly between patients treated and not treated with amiodarone, suggesting that VNC also subtracts iodine from iodine-containing amiodarone accumulation in the liver. These initial findings were then verified in a phantom scan, in which VNC of TNC were able to subtract attenuation of iodine-containing amiodarone. Further, the results from our first analysis could be confirmed in an additional patient cohort, for which, unlike to our initial patient cohort, also VNC reconstructions of TNC images were available. In line with our initial results VNC was also able to reduce liver attenuation and LAI in patients treated with amiodarone to a level comparable to patients not treated with amiodarone.

Our patient cohort only included five patients with reduced liver attenuation and LAI indicating liver steatosis, therefore we could not add a further pathology focused analysis on liver steatosis. Still, our data suggests that VNC could be relevant in these patients. In the group of amiodarone patients, VNC led to a mean decrease of LAI of around 10 HU compared to TNC, potentially representing artificially high attenuation in TNC from the iodine in amiodarone. This difference can be relevant in amiodarone patients when it obscures potential liver damage reflected by liver steatosis^1,7,8^. The LAI is predictive for a liver fat fraction of >30% and image-based diagnosis of liver steatosis when the difference is less than −10 HU^39–41^, hence a mean difference of 10 HU could possibly lead to many misclassified patients. Due to its toxicity amiodarone is also known to independently induce steatohepatitis^30^ which can then result in liver cirrhosis, drastically increasing risk of mortality^1,7,8^. Further, liver steatosis, that is caused for other reasons than amiodarone, is common in a general population with non-alcoholic liver steatosis as its most common subtype and an incidence of 15% in general population^24,42^. A severe liver fat fraction of ≥30% is considered to have relevant clinical implications^43,44^ but could be obscured in amiodarone patients due to amiodarone accumulation in liver^24,45,46^. As described above, VNC can also overestimate attenuation of fat; therefore, it needs to be considered that VNC can negatively affect CT attenuation-based diagnosis of liver steatosis in CT imaging^24^.

For patients with increased liver attenuation undergoing amiodarone treatment, material decomposition and TNC might give radiologists and clinicians more confidence to identify amiodarone as the cause of increased liver attenuation^7,28,29^, potentially allowing for more precise monitoring of liver attenuation and earlier adaption of drug treatment. Identification and quantification of amiodarone in the liver might also be useful as additional guidance for amiodarone treatment, as tissue levels have been suggested to be useful to anticipate toxic liver damage and treatment efficacy^7,18,29^. Differences in liver attenuation between TNC and VNC could be used as a measure to quantify amiodarone accumulation in the liver^21,47^. Thus, spectral imaging might serve as a quantitative biomarker for treatment monitoring in patients before and/or under amiodarone with and with-out fatty liver disease, which should be evaluated in future studies.

The study has some limitations that need to be considered. Spectral imaging data for the unenhanced chest acquisition (TNC) is not routinely saved at our institution, therefore creation of VNC images from this phase was not possible in the initial cohort. However, to address this shortcoming we changed our image reconstruction protocol and included an additional cohort in which VNC was also reconstructed from TNC scans; however, we refrained from conducting a pooled analysis as in the initial patient group VNC was performed on contrast enhanced CT scans and in the additional patient group VNC was performed on unenhanced scans. In this cohort we were able to show that VNC of TNC can reliably reduce increased liver density due to amiodarone deposition. As frequently applied in comparable studies, we used ROI-based measurements of mean attenuation^22–25,35^. Still, a volumetric assessment of attenuation covering an entire organ might be less susceptible for any measuring bias. To minimize mismeasurement, we used two ROI for spleen and four ROI for the liver with a standardized size and averaged the results. Further, we placed ROIs in the same anatomical locations in each image set with the help of screenshots of the initial placement.

In conclusion, patients treated with amiodarone showed significantly higher liver attenuation in TNC. In patients not treated with amiodarone VNC accurately subtracted contrast-agent related iodine in contrast-enhanced examinations. Also, VNC depicted comparable liver attenuation in patients treated and untreated with amiodarone indicating that VNC subtracted iodine resulting contrast injection as well as from hepatic amiodarone deposits.

## Materials and Methods

**Figure 3:**
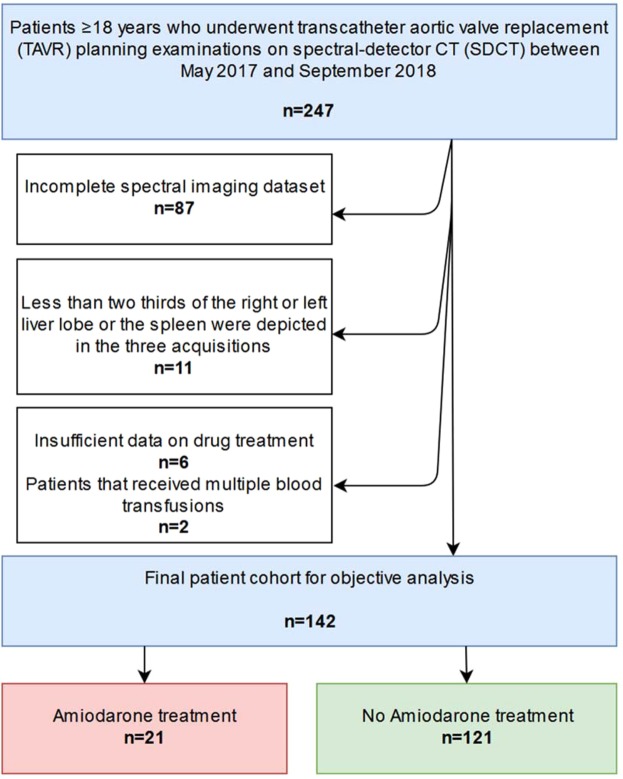
Flowchart demonstrates study inclusion process undergone by patients with transcatheter aortic valve replacement (TAVR) examinations on spectral-detector CT (SDCT).

The institutional-review-board approved this retrospectively designed study. The standards of the Health-Insurance-Portability- and Accountability-Act were followed. Under Code-of-Federal-Regulations (title 45, §46.116d) informed written consent was waived. Following criteria for patient inclusion were applied: (i) patient age ≥18 years, (ii) patients with severe aortic stenosis receiving a TAVR planning examinations on a clinical spectral-detector CT system (IQon, Philips Healthcare) between May 2017 and September 2018, (iii) complete imaging data set, including the following examinations in the given order, unenhanced chest [TNC], CT angiography chest [CTA-Chest], and CTA abdomen [CTA-Abdomen]), (iv) at least two thirds of the spleen as well of the liver depicted in all three acquisitions, (v) sufficient data available on drug and/or amiodarone treatment, respectively, and (v) no reports of multiple blood transfusions, hemochromatosis, Wilson’s and/or glycogen storage diseases in patient history to exclude possible confounder for elevated liver attenuation^48^, (Fig. 3). TNC chest examinations were executed to perform aortic valve calcium scoring prior to TAVR procedure. This resulted in total of 142 patients included in the final analysis. Patients that were treated with amiodarone at time of the TAVR examination were grouped in the amiodarone cohort (n = 21), patients without amiodarone treatment were grouped in the no amiodarone treatment cohort (n = 121). Body mass index, history of smoking, and presence of diabetes, arterial hypertension, abnormal liver laboratory tests, as well as chronic liver disease were recorded for each patient to detect any potential confounders between the two groups.

### Imaging protocol

Detailed scan parameters can be seen in Table 4 for the unenhanced chest scan, CTA-Chest and CTA-Abdomen. For CTA-Chest examinations, 25 ml of iodinated contrast agent (Optiray 350, Guerbet) were administered through an antecubital vein. A retrospective ECG-gated helical scan was initiated using bolus-tracking: a region-of-interest (ROI) was placed in the left atrium and scans were initiated after an attenuation threshold of 60 Hounsfield units (HU) was reached. For CTA-Abdomen, another 25 ml of contrast agent were administered 120 seconds after the chest scan. The scan was started using bolus-tracking with a ROI in the descending aorta when attenuation increased 10 HU over the baseline. This threshold was chosen, as contrast in vessels from the previous scan was still present but differing between patients due to individual differences in circulation. After testing we therefore decided to use an increase of 10 HU over the baseline instead of an absolute threshold. Iterative reconstructions were performed for unenhanced chest scan (TNC), CTA-Chest and CTA-Abdomen. VNC were created from CTA-Chest and CTA-Abdomen data using the vendor’s dedicated material decomposition and spectral reconstruction algorithms (Spectral B, level 3, Philips Healthcare).

**Table 4:** Scan parameters.

	Tube voltage	Tube current/Dose right index (DRI)	Collimation	Rotation Time	Pitch	Gating	Slice thickness	Conventional iterative reconstructions	Spectral reconstructions
Unenhanced Chest	120 kVp	Automatically adapted to patient size*/11	64 × 0.625 mm	0.33 seconds	n/a	Prospectively ECG-triggered axial scan (Step & Shoot)	2 mm and 50% overlap	iDose 4, level 3, Philips Healthcare	n/a
CT Angiography of the Chest	120 kVp	Automatically adapted to patient size*/18	64 × 0.625 mm	0.33 seconds	0.2	Retrospective ECG-gated helical scan	2 mm and 50% overlap (n = 110), 0.9 mm (n = 32) and 50% overlap	iDose 4, level 0, Philips Healthcare	Spectral B, level 3, Philips Healthcare
CT Angiography of the Abdomen	120 kVp	Automatically adapted to patient size* and modulated based on patient anatomy^†^/14	64 × 0.625 mm	0.5 seconds	1.02	n/a	2 mm and 50% overlap	iDose 4, level 3, Philips Healthcare	Spectral B, level 3, Philips Healthcare

*Achieved using DoseRight (Philips Healthcare), ^†^Achieved using 3D DOM (Philips Healthcare).

### Additional patient cohort

To verify if VNC can truly remove attenuation from amiodarone accumulation of the liver we included additional TAVR patients (n = 145) receiving the protocol from above and outlined in Table 4 between December 2018 and June 2019. For these patients, reconstructions of VNC images from the TNC scans were available. Eleven of these patients received amiodarone treatment (supplementary Table 1).

### Objective analysis

Circular ROIs of at least 500 mm^2^ were placed on axial images to measure the mean attenuation in HU by a radiologist with four years of experience in abdominal imaging. ROI were first drawn in CTA-Abdomen (because anatomical identification and delineation of structures was most accurate here) and subsequently were transferred to TNC and CTA-Chest as well as VNC-Chest and -Abdomen using the vendor’s proprietary image viewer (IntelliSpace Portal v9, Philips Healthcare). ROI positions could differ for image sets derived from different acquisitions due to respiratory motion. To minimize any mismeasurements from different ROI locations, screenshots of each ROI in CTA-Abdomen images were used to match placement in the other image sets. Two ROIs each were placed in the right and left liver lobe as well as in the spleen. The results of the four ROIs from the right and left liver lobes were averaged to obtain results for the entire liver. The two ROIs of the spleen were also averaged. The liver attenuation index was calculated as the difference of attenuation between liver and spleen^13,41^.

### Phantom measurements

In addition, we also performed a phantom analysis using falcon tubes placed in a non-anthropomorphic water phantom using a helical scan with the scan parameters from the unenhanced scan of the chest. The falcon tubes were filled with 30 g of minced pig’s liver; once without any additions and once with amiodarone hydrochloride aiming for a concentration of 0.2 mg I/ml^49^. The water phantom with the falcon tubes were scanned three times each. Three ROIs on different levels were placed centered in each of the two falcon tubes for each of the three scans and then averaged.

### Statistical analysis

Statistical analysis was conducted using JMP Software (V14, SAS Institute). Quantitative results are presented as mean ± SD. Shapiro-Wilk test was used to test for normal distribution. Wilcoxon rank-sum test was used to test for any difference of attenuation in between patients treated and not treated with amiodarone. Mann-Whitney-U test was used to test for any difference of attenuation between TNC and VNC. Statistical significance was defined as p < 0.05. To estimate number of patients to evaluate if VNC can subtract attenuation of iodine from amiodarone we conducted a power analysis, using G*Power (v. 3.1.9.4)^50^. According to the literature, the mean attenuation of liver was assumed to be 60 HU^51^ with a standard deviation of approximately 6 HU^52^. We estimated that mean accumulation of amiodarone in the liver should result in an increase of attenuation around 5 HU, resulting in an estimated effect size of 0.83. We aimed for a power of at least 0.8. Assuming a distribution between groups of 1:6 (amiodarone vs. no amiodarone treatment), our sample size should be at least 98 patients (84 patients no amiodarone, 14 patients with amiodarone treatment).

## Supplementary information

Supplementary Data.

## Data Availability

The datasets generated during and/or analyzed during the current study are available from the corresponding author on reasonable request.
