# Supplementary material for: Iodine accumulation of the liver in patients treated with amiodarone can be unmasked using material decomposition from multiphase spectral-detector CT
Source: Sci Rep. 2020 Apr 24;10:6994. doi: 10.1038/s41598-020-64002-7 (PMC7181843; doi:10.1038/s41598-020-64002-7)

Iodine accumulation of the liver in patients treated with amiodarone can be unmasked using material decomposition from multiphase spectral-detector CT

Authors:

Kai Roman Laukamp[1,2,3, *], Simon Lennartz[3,4], Ahmad Hashmi[1,2], Markus Obmann[5], Vivian Ho[1,2], Nils Große Hokamp[1,2,3], Frank Philipp Graner[1,2], Robert Gilkeson[1,2], Thorsten Persigehl[3], Amit Gupta[1,2], Nikhil Ramaiya[1,2]

Affiliation:

[1]University Hospitals Cleveland Medical Center, Department of Radiology, Cleveland, OH, USA
[2]Case Western Reserve University, Department of Radiology, Cleveland, OH, USA
[3]University Hospital Cologne, Institute for Diagnostic and Interventional Radiology, Cologne, Germany
[4]Department of Radiology, Massachusetts General Hospital, 55 Fruit St, White 270, Boston, MA 02114
[5]University Hospital Basel, Department of Radiology and Nuclear Medicine, Basel, Switzerland
*Corresponding author

Corresponding author:

Dr. Kai Roman Laukamp
Department of Radiology, University Hospitals Cleveland Medical Center
11000 Euclid Ave
Cleveland, OH 44106
telephone: +1 216 844 7519
fax: + 1 216 983 0798
kai.laukamp@UHhospitals.org
kai.laukamp@uk-koeln.de

Supplementary Table 1 - Amiodarone patients of the additional patient group

Patient	Gender	Age	Amiodarone dose per day [mg]	Treatment duration [months]
1	female	93	200	69
2	male	77	200	2
3	female	61	200	27
4	female	79	200	6
5	male	76	200	4
6	female	95	200	3
7	male	77	200	4
8	male	57	100	5
9	male	76	200	3
10	female	78	200	3
11	female	81	200	6

Supplementary Figure 1: Box-plot diagram displaying attenuation in liver and liver attenuation index (LAI) in **A)** true non-contrast (TNC), **B)** virtual non-contrast (VNC) from the unenhanced chest scan in the additional patient group.

A), Patients receiving amiodarone treatment showed significantly higher liver attenuation and LAI indicating that amiodarone accumulation in the liver artificially increased attenuation values of the liver.

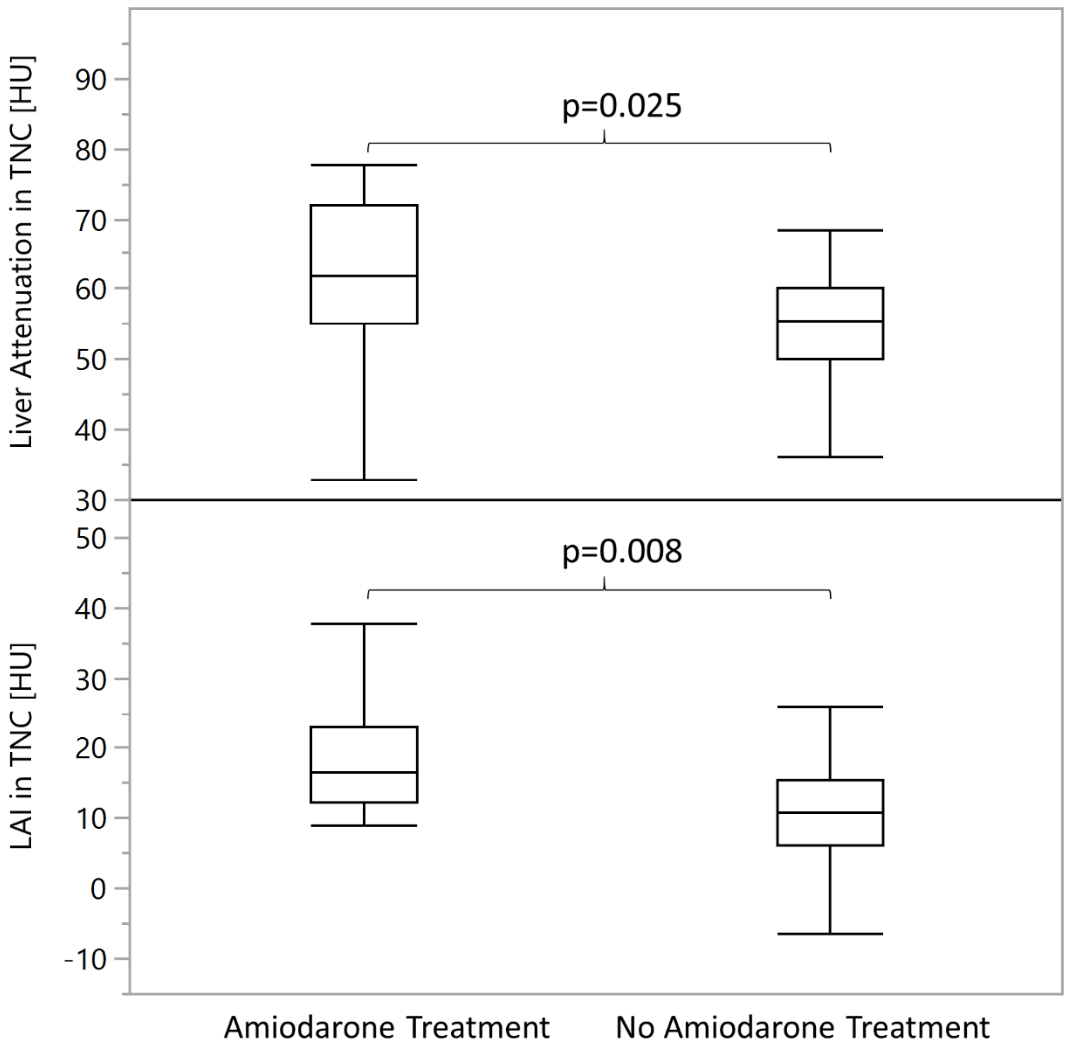

B), Contrary to TNC, liver attenuation and LAI in VNC images of patients treated with amiodarone were comparable without significant differences to patients that have not been treated with amiodarone indicating that VNC is also able to subtract the iodine from amiodarone accumulated in the liver.

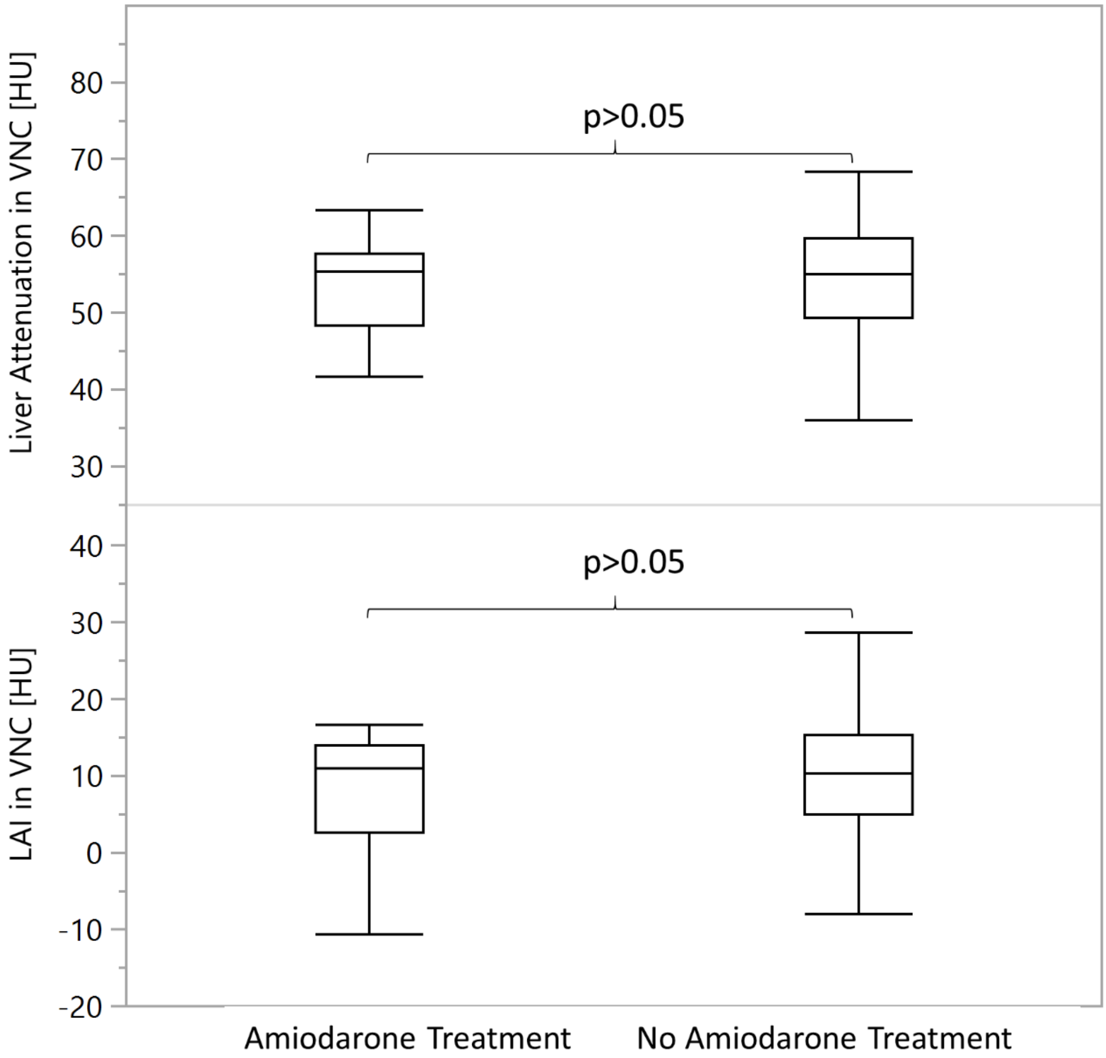

Supplement: Supplementary file 1 — Supplementary Data. [file 41598_2020_64002_MOESM1_ESM.pdf]
